# Postretirement Leisure Participation in a Collective Culture: Experiences and Perspectives of Older Adults and Family Members in an Indian Metropolis

**DOI:** 10.1007/s10823-025-09528-x

**Published:** 2025-04-05

**Authors:** Shreya S. Kamath, Sebestina Anita Dsouza

**Affiliations:** 1 Department of Occupational Therapy, Manipal College of Health Professions, Manipal Academy of Higher Education, Manipal, India; 2 Centre for Studies on Healthy Aging, Manipal Academy of Higher Education, Manipal, India

**Keywords:** Collectivistic culture, Family, Leisure, India, Retirement, Older adults

## Abstract

Leisure is essential to support quality of life following retirement. In India, there is limited research on the leisure participation of older adults. In collective cultures such as India, family members may also influence older adults’ leisure participation. Therefore, the present study aimed to understand the experiences and perspectives of Indian older adults and their family members regarding leisure participation following retirement. The present study used an interpretative phenomenological approach with in-depth interviews. The participants were eight dyads of older adults and their family members residing in Mumbai, an Indian metropolis. The findings suggest that older adults described activities that gave them satisfaction as leisure, such as preretirement hobbies, work-related activities, volunteering, and activities for and with family, including household chores. The study also identified personal, familial, and contextual factors influencing older adults’ leisure participation. The findings of this study can inform the provision of contextually relevant interventions to support meaningful leisure participation in older adults and healthy aging.

## Introduction

Retirement is a salient life event and a milestone in an individual’s life. Following retirement, older adults’ engagement in leisure activities increases (Gauthier & Smeeding, 2010; Henning et al., 2021) and is associated with positive benefits such as improved health, life satisfaction, and quality of life (Cha, 2018; Sala et al., 2019; Nimrod & Shrira, 2016; Badami & Yenagi, 2015). In India, the age of retirement is usually between 58 and 65 years of age in the public and private sectors and is not specified for the unorganized sector. Reports suggest that the percentage of retired older adults (aged 60 years and above), i.e., those not in the labor force, increased from 60.85% in 2011–12 to 68.95% in 2018–2019 (National Statistical Office [NSO], 2021). Moreover, life expectancy has increased (World Bank, n.d.). Consequently, the expected number of years in retirement increased from 9.3 years for men and 13.6 years for women in 2008 to 11.2 years for men and 17.1 years for women in 2018 (Organization for Economic Co-operation and Development [OECD], 2021). The increasing number of years in retirement make it imperative to address retired older adults’ health and well-being, including their leisure interests. Leisure activities are a relatively inexpensive and feasible approach for disability prevention and health promotion in older adults (Hutchinson & Nimrod, 2012; Chang et al., 2014), especially in low- and middle-income countries such as India.

## The Leisure of Indian Retirees

Studies across cultures have reported that older adults tend to pursue leisure interests acquired when they are young (Minhat et al., 2013; Agahi et al., 2006; Wanka, 2020). While leisure patterns remain relatively stable across the life span, during later life, individuals experience losses in informal, formal and physical leisure, especially outdoor leisure. These losses were attributed to diminishing health rather than age and to factors such as retirement and changes in marital status (Janke et al., 2006). There are very few studies on the leisure activities of Indian older adults. Badami and Yenagi (2015), in their cross-sectional study in southern India, identified various leisure activities of older adults, such as going for walks, engaging in religious activities, sports/games, gardening or reading, volunteering or community activities, watching television, listening to music, sleeping and working part/full time. However, older adults’ leisure choices and participation may be influenced by several contextual factors that need to be understood.

Culture and nationality influence leisure choices, participation, and satisfaction (Iwasaki, 2006). For example, American adults identified nine categories of retirement goals, including exploration, self-oriented goals, leisure, and financial stability (Hershey et al., 2002). In contrast, Indians have fewer retirement goals and prioritized financial stability, contributing to others or acquiring possession over leisure and exploration (Gupta & Hershey, 2016). Additionally, leisure choices and experiences depend on the meaning ascribed to leisure, which may differ across cultures. The conventional concept of leisure is drawn primarily from the Western world, especially North America and Europe (Purrington & Hickerson, 2013). Leisure represents segmented portions of time or activity or fleeting states of mind and is described as having three essential components: (1) time free from work and daily chores; (2) activities that are free of obligations; and (3) providing a positive and pleasant experience that may be subjective (Henning et al., 2021; Kleiber & Nimrod, 2009; Huang & Li, 2019). These Western-oriented concepts of leisure may not reflect the concept of leisure in non-Western cultures. Studies in Asian cultures have reported that freedom may not be an essential part of the leisure experience (Walker & Wang, 2008). For example, Chinese older adults have subjective interpretations of leisure (Huang & Li, 2019). There is limited information on the meanings ascribed to leisure by older Indians.

Furthermore, studies suggest that the influence of leisure participation on older adults’ well-being may be enhanced or inhibited by family and social dynamics, necessitating an understanding of the family and community context (Cuenca et al., 2014). Like most Asian countries, India is described as collectivistic in nature, with individuals being seen as rooted within their group identity. Collectivistic cultures promote interdependence and cooperation, and the family is the focal point or the basic social unit (Chadda & Deb, 2013). The living arrangement may further impact familial influences. Traditionally, in Indian society, older adults live with their children and/or other family members. These are known as joint or extended families, cohabitation, or coresidential living arrangements. In the present paper, these families are referred to as joint families. Members of the joint family include children, grandchildren, or other relationships and often involve several generations living together (Rajan & Kumar, 2003). Living arrangements have changed considerably over the last several decades due to globalization and urbanization. In 2017–2018, 81.7% of older adults lived in joint families in India, suggesting that joint families continue to be the preferred living arrangement. The meaning and values ascribed to leisure differ across generations (Twenge et al., 2010), and these differences may impact older adults living in joint families. Other alternative living arrangements are also reported wherein families live separately but close to each other, such as in the same building, neighbourhood or town (NSO, 2021). Whether living together or separately, interdependence is a common feature of collectivistic societies (Chadda & Deb, 2013), and family members may invariably influence older adults’ leisure participation.

Indian families are also described as patriarchal in structure, wherein roles, responsibility, control, and distribution of resources within the family are determined by age, gender, and generation (Sonawat, 2001). Gender-based differences have been reported in leisure participation, with women being more involved in home-bound caring or nurturing roles, whereas men prefer outdoor, physical, and productive activities across cultures, and men have more leisure time and more access to leisure than women do. These differences are said to depict the sexual division of labor in societies and traditional gender stereotypes (Tsai, 2010; Jaumot-Pascual et al., 2018). These differences may be more prevalent and deep-rooted in patriarchal societies such as India (Khurana, 2018), and household chores may constrain the leisure participation of older women (Bhangaokar et al., 2021).

Other contextual factors influencing leisure choices and participation include the availability of resources, access, literacy, health, etc., which differ between rural and urban areas. They are unique and need to be studied separately. There is limited research on the contextual factors influencing the leisure of Indian older adults. The present study intended to address this gap and aimed to understand the leisure participation of Indian older adults in an urban setting. The primary research questions were as follows:

1. What are the experiences and perspectives of urban Indian older adults regarding leisure following retirement?
2. What is the influence of family members on the leisure participation of urban older adults?
3. What are the experiences and perspectives of family members regarding the leisure participation of their older family members following retirement?

## Methods

### Study Design

The study used an interpretative phenomenological approach (IPA) that provides a deeper and context-specific understanding of the participants’ personal experiences. It allows the researcher to interpret the participant’s experiences on the basis of their values, beliefs, experiences, and theoretical knowledge, thus enabling higher-order interpretations (Cronin-Davis et al., 2009; Alase, 2017; Smith & Fieldsend, 2021; Fischer, 2009). The IPA involves a double hermeneutic approach in which researchers attempt to integrate the ‘emic’ and etic’ perspectives. The former (emic) aims to understand the phenomenon as experienced by the participants. The latter (etic) involves the researcher attempting to interpret participants’ experiences. In IPA researchers navigate between layers of interpretation to develop a multilayered narrative of possible meanings (Eatough & Smith, 2017).

Another important aspect of IPA is ideography, wherein the researchers attempt to understand the lived experience of each participant before making general statements. IPA requires the researchers to be mindful of their preconceived beliefs or assumptions through reflective practices, including bracketing (Cronin-Davis et al., 2009; Alase, 2017; Smith & Fieldsend, 2021; Fischer, 2009). Reflexivity encompasses the entire research process and refers to a ‘set of continuous, collaborative, and multifaceted practices through which researchers self-consciously critique, appraise, and evaluate how their subjectivity and context influence the research processes’ (Olmos-Vega et al., 2022). Bracketing typically refers to researchers identifying their vested interests, personal experiences, cultural background, and assumptions, which could influence their interpretation during data analysis (Fischer, 2009).

The authors share similarities with the study participants with respect to the languages spoken, the sociocultural environment and the phenomenon being investigated. The primary author lived in a joint family with grandparents in an apartment in Mumbai. The corresponding author lived in a nuclear family in an independent house in Mumbai until adulthood and later lived in a joint family with older parents-in-law in an independent house in another Indian city. Furthermore, the authors concede that their professional training is drawn mainly from Western literature. The authors acknowledge that these factors may have influenced the study.

### Participants

The study participants were dyads of older adults and family members who had adequate comprehension, hearing, and communication abilities; were independent in their daily activities; and spoke Hindi, Marathi, or English. The older participants included individuals aged 60 years and above who had retired from formal or informal employment upon reaching superannuation or by personal choice. The family member was an adult aged 18 years and above who was familiar with the older participant’s routines and daily activities, irrespective of the living arrangement. The study excluded participants with an acute illness at the time of recruitment or any self-reported disabling physical, cognitive or psychiatric disorders. For recruitment, participants were purposively selected with the help of a local informal network, contacts of the investigators, and snowballing.

### Study Setting

The study was based in Mumbai, an Indian metropolis, owing to the authors’ familiarity with the city, culture, language, and convenience. It is the capital of Maharashtra state and an urban agglomerate. Owing to its strategic location and connectivity, it is a leading industrial, manufacturing, and commercial center. It is the financial capital of India and the most urban Indian city. Owing to its high degree of migration, Mumbai is the most populous and diverse Indian city and ranks fourth globally. It has a literacy rate of 89.73% (World Population Review, n.d.). In 2011, Mumbai had 7,67,618 older adults aged 60 years and above. Approximately 16.78% of households with four members and above had at least one older adult (Census of India, 2011). Mumbai is known for its fast pace of life and is commonly described as a city that never sleeps. Marathi is the dominant regional language, followed by the official languages of Hindi and English. While Hindi and Marathi are different languages, they are both classified under the Indo-Aryan family of Indian languages and have the same origin and similar scripts, words and meanings (Cardona, n.d.).

### Study Procedure

The study was approved by the institutional ethics committee of Kasturba Medical College and Kasturba Hospital [IEC:484/2018] and was conducted between August 2018 and March 2019. In-depth interviews were conducted via a semi structured interview guide developed through iterative discussions among the authors. The interview guide was based on the research question and did not entail any preexisting theories or preconceived hypotheses, ideas, or concepts about the phenomenon (Creswell, 2003; Alase, 2017). The interview guide was not prescriptive and used open-ended questions to understand the participants’ lived experiences and perspectives.

The main questions for the older adults were as follows: (1) Could you explain what you mean or understand about the term leisure? (2) Could you describe your experience of engaging in or participating in leisure activities following retirement? The main questions for family members of older adults were as follows: (1) Could you explain what you mean or understand about the term leisure? (2) Could you describe the leisure activities of your older relative following retirement? (3) What are your opinions regarding their leisure participation? The questions progressed from general to specific and used probes to stimulate discussions. Some examples include, ‘You mentioned… Please explain that further. Could you tell me more about that?’

The authors developed the interview guides in English. The primary author translated the interview guide to Hindi and Marathi with the consultation of a bilingual expert as needed. In Hindi, avkasha is the most immediate word for free time when there is no work, and one can do things they enjoy (Collins, n.d.). Leisure was explained as *avakasha* in Hindi with synonyms such as *fursat* and *khali samay* and *fursati chya veli* in Marathi. The primary author was proficient in all three languages.

The primary author contacted potential participants by telephone, briefed them about the study, and scheduled in-person meetings at their homes. Details of age, education, living arrangement, past worker role, time since retirement, health, and self-reported socioeconomic status (SES) were collected. Older adults and their family members meeting the study criteria provided written consent for their participation in the study. The primary author individually interviewed the participants in person on the basis of their convenience and language preferences at their homes. All interviews were audio-recorded and lasted for 40–50 min. The primary author took notes and recorded observations within two to three hours after each interview.

The interviews were then deidentified, given pseudonames, transcribed, and translated into English within two to three days. The primary author reviewed the recordings and transcripts several times and consulted bilingual experts for uncommon and colloquial words or phrases to ensure semantic and cultural relevance in translation (Regmi et al., 2010). For each Indian language, two bilingual experts were consulted. The experts were language teachers at schools and colleges in the study setting and were thus familiar with the participants’ linguistic and cultural context. The experts back-translated two initial transcripts each of Hindi and Marathi. The forward and back translations were found to be satisfactory. For the remaining transcripts, only forward translation was performed, and experts were consulted as necessary because of limited resources (Regmi et al., 2010). The corresponding author verified the transcribed data with the recordings to ensure accuracy. The deidentified transcripts were then printed. Throughout the study, the authors maintained the participants’ confidentiality and anonymity. After each interview, the authors read the transcript, referred to the field notes, and reflected on the findings. The authors suspended data collection after recruiting 16 participants, a manageable number for in-depth analysis.

### Data Analysis

In line with IPA, the authors incorporated the principles of bracketing, reflexivity, and peer critique throughout the analysis process (Peat et al., 2019; Smith & Fieldsend, 2021; Fischer, 2009). The authors identified and wrote down their assumptions and beliefs about the phenomenon from their personal experience of living in a joint or nuclear family, role in the family (grandchild/child/spouse), living in the study setting, personal factors (e.g., age, gender, generation), professional background, or from the literature reviewed. For example, ‘Leisure is an activity that you do in your free time and does not include work, household chores, and other obligatory activities; older adults’ preferences are usually prioritized and respected in joint families; outdoor leisure activities of older adults in Mumbai may be affected by poor road conditions, crowded places and traffic; older women prefer home-oriented leisure activities; and older adults pursue familiar leisure interests following retirement’. With emerging data, these assumptions of the phenomenon and interpretations were reexamined. The authors made conscious efforts to be mindful of their assumptions and avoid imposing them on the participants’ narratives. The authors engaged in reflexivity throughout the study through self-reflection (writing) and collaborative reflection (discussions with each other) (Peat et al., 2019; Smith & Fieldsend, 2021; Fischer, 2009).

The analysis involved manual handling of the transcripts and commenced from the first interview. The authors independently read each transcript carefully several times to familiarize themselves with the participant’s narrative of the phenomenon as experienced in his or her context. Next, the authors read each transcript line-by-line. The authors underlined salient statements, phrases, and quotes describing the participants’ perspectives of the phenomena and made notes or comments (descriptive, linguistic, & conceptual) in the margins. Next, through an interpretive lens, these initial comments were converted into concise statements and then further condensed into codes (phrases of three to five words) that best represented the statement without diminishing its meaning. The codes were then grouped into categories and themes on the basis of similarities, connections, and differences. The authors compiled a list of themes and categories with supporting quotes that represented the participants’ unique experience of the phenomenon. This process was repeated for each transcript. The authors then examined the themes of all participants for patterns of commonalities and differences across participants and identified overarching themes and categories. Discrepancies between the authors regarding the codes, categories, themes, and interpretations were resolved through discussions. The findings were presented at a departmental meeting that was attended by several colleagues, including two senior faculty members with expertise in qualitative research. Further discussions ensued, and a final list of themes and categories was developed for the study (Peat et al., 2019; Smith & Fieldsend, 2021; Fischer, 2009).

## Results

Twelve older adults were initially identified as potential participants. The authors excluded four older adults, as two were working full-time, one had hearing impairment, and one had cognitive impairments. Finally, eight older adults and eight family members participated in the study. The age of the older adults ranged from 62 to 74 years. Four interviews were conducted in Marathi, three in Hindi, and one in English. Tables 1 and 2 describe the older adults’ characteristics and living arrangements. They rated their health as good to very good and received a pension.

**Table 1:** Characteristics of older participants

Older adult	Age (years)	Gender	Pre-retirement occupation	Years since retirement	Socio-economic status	
Veer	74	Male	Chief security officer	16 years	Middle	
Anasuya	63	Female	Teacher	5 years	Upper	
Vishwas	63	Male	Deputy manager	3 years	Middle	
Udav	63	Male	Vice president	3 years	Upper	
Arti	63	Female	Teacher	2 years	Middle	
Suhash	66	Male	Bank manager	6 years	Upper	
Pankaj	62	Male	Lead Manager	2 years	Middle	
Vinayak	72	Male	College Registrar	12 years	Middle	

**Table 2:** Living arrangements of the older participants

Older adult	Living arrangement	Housing	Family size	No. of generations
Veer	Joint	2-BHK	5	3
Anasuya	Joint	2 adjacent 1-BHK	6	3
Vishwas	Joint	1-BHK	3	2
Udav	Joint	2–BHK	3	2
Arti	Nuclear	2-BHK	2	1
Suhas	Nuclear	1-BHK	2	1
Pankaj	Joint	2-BHK	5	3
Vinayak	Joint	2-BHK	6	3

*BHK* Bedroom, Hall, and Kitchen

Most of the participants lived in joint families. Anasuya lived with her husband in an apartment with one bedroom, a hall or living room, and a kitchen (BHK). Her son lived next door in a similar 1-BHK. The two individual apartments had a connecting door and functioned as one house. They performed most daily activities together as a family, such as cooking, dining, watching television, and celebrating festivals. Anasuya described their living arrangement as a joint family. Another participant, Aarti, lived with her spouse in a 2-BHK apartment next to her son and his family. The two families did things independently but interacted daily and were familiar with each other’s routines. She described her living arrangement as a nuclear family. The age of the family members ranged from 18 to 63 years. Two family members were interviewed in Hindi, and the rest were interviewed in English. Table 3 describes the family members’ characteristics.

**Table 3:** Characteristics of the participating family member

Family member		Age (years)	Occupation	Relation with older participant
Shirish	30		Lawyer	Son of Veer
Gauri	34		Homemaker	Daughter-in-law of Aarti
Ashawari	59		Homemaker	Wife of Vishwas
Ujaya	62		Teacher	Wife of Udav
Rashika	39		Manager	Daughter-in-law of Anasuya
Shobha	63		Homemaker	Wife of Suhas
Aditya	29		Engineer	Son of Pankaj
Siddhesh,	18		Student	Grandson of Vinayak

The study findings were organized into two broad themes: activities that provide satisfaction are leisure for older adults and factors influencing older adults’ leisure participation.

### Theme 1: Activities that Provide Satisfaction Are Leisure for Older Adults

This theme describes older participants’ leisure interests following retirement. Their narratives indicate that leisure activities were something they found pleasure in doing, which gave them satisfaction. The identified leisure activities were categorized into preretirement hobbies, work-related activities, and family-oriented activities.

#### Preretirement Hobbies and Work-Related Interests

Following retirement, with more time on their hands, older participants took the opportunity to catch up with their former leisure interests or hobbies that were left untouched due to work commitments. Aarti said, *“I get time now (after retirement…I like to make different types of foodstuffs.”* They were inclined to pursue their preretirement interests as leisure. Older women engaged in leisure activities such as cooking, watching television and online devotional or cooking videos, going for walks, and doing yoga. Older men identified walking, reading the newspaper, and watching television as their leisure interests.

The study findings also suggest that older adults prefer to continue with their work roles in some way or another way. Some continued to offer their professional expertise as volunteers, and some found work-related alternatives. They described these activities as leisure, as they derived satisfaction from these activities. For example, Anasuya, a retired teacher, continued to do some work for her school.

My school place is nearby, and even though I am retired. I still have lots of work (there). I am interested in doing it, so I go to school sometimes in the afternoon. Therefore,, if there is something to write or to be done, then I do that, or they sometimes send it over.

Similarly, Udav started working towards his lifelong desire to develop new technology in his field using new ideas owing to the vast amount of time at his disposal. He said, “*While I was working…so during that period I did not get any time for these research activities*,* so now I have started doing this research.*” Vinayak, who worked as a registrar in a renowned college for approximately 40 years, expressed his reluctance to leave behind his worker role: “*In the beginning (postretirement)*,* I used to feel that I have not retired only. I was very much interested in work*,* so I started working for my society as a treasurer*.”

#### Family-Oriented Activities

Older adults found it satisfying to perform activities with their family or for their family. Aarti explained her way of spending more time with her husband by turning a solitary leisure activity into a couple-oriented activity: “*My husband and I read the newspaper together and then discuss it.*” Although Veer liked outdoor leisure activities, he expressed his happiness in returning home and spending some time with his wife.

After roaming around (outside), I come home and chat with my wife. That used to not happen before because if I go (for work), then I would be doing duty for eight days in a row in an emergency situation. Therefore, that was not possible before. So chatting and all happen while I am sitting at home now.

The older adults in this study valued the togetherness and pleasure they experienced in performing leisure activities with their family members. Anasuya described, “*We sit together here…and we get together and watch…if the TV is on then*,* the whole family watches together*.” Older participants scheduled leisure activities on the basis of their family members’ preferences and availability to ensure participation.

We see the review in the newspaper…or on internet, we see the review…we decide…with my son…because he is very particular about that…so we decide with him…when he says yes…we will go for this movie, then we will go at any time, even for the last show. (Udav)

Older adults also engaged in activities such as household chores for the benefit of their family members and described it as leisure, as it gave them satisfaction.

I call them (buying vegetables) leisure… not work. If my family says that they want these things, then I go and get them. (Vinayak).

There are two kinds of walking he (Vinayak) does. Like one is for work, he travels a lot, and because of that, he walks a lot. However, the other kind of walking like sometimes he goes for a leisurely stroll. Again, it is only sometimes. However, he goes for a leisurely stroll with my grandmother…like recently my grandmother had a health problem and because that doctor suggested that she should walk more, she should exercise more. So that has come across as an excuse to walk. (Siddhesh).

### Theme 2: Factors Influencing Older Adults’ Leisure Participation

#### Personal Factors

Health issues influence older adults’ leisure participation. While some reduced their frequency of participation, others had to discontinue or relinquish their leisure activities.

Only restrictions I get are due to my physical issues because of this knee joint pain. Because of this, some types of yoga-like Vajra Shan and all I cannot do, and that is why I do not like to go there (yoga class). (Arti)

I do not read novels and all because they have small letters…so that is why it is difficult for me to read. Even if I wear spectacles and all, it is difficult to read. (Vinayak)

Older adults were also concerned about their safety and were not confident in their ability to participate in complex leisure activities by themselves.

Therefore, if I am not safe, then I should not go (to parks). (Veer)

I motivate him (Vinayak) to travel. Like in the past few months, I am telling him that we should book tickets for Sri Lanka. Therefore, he says that he cannot go…he cannot go alone. (Shirish, son of Vinayak.)

Older adults, especially those who considered working or being productive leisure activities, were unwilling to participate in more ‘leisurely’ activities, even when encouraged by their family members. Family members attributed this unwillingness to the older person’s inflexibility, personality, lack of motivation, or resistance to change.

It should come from themselves…change in yourself. If you think you want to go out, you want to do walking, then you should get up from the sofa and at least walk in the home…but you have to start on your own… if a social worker or someone says that I have made joggers park for you, go there (laughs). Unless you feel on your own to do something, then no one can do anything. (Ujaya, wife of Udav)

He has a stagnant mindset. Therefore, he does not like new information or new things. There are groups or clubs for retired people. Therefore, I told him to go for it, but it was not happening. He keeps on saying that there is no need for that. (Shirish, son of Veer)

The willingness to adopt technology was another factor that supported leisure participation. Anasuya said, “*So that we are in line with the time…it is necessary to do that (use technology)*,* and I enjoy it…to watch and all…mostly cooking videos…something new…and meditation videos come on YouTube… so I watch that also.*”

#### Intergenerational Differences in Leisure Perspectives in the Family

The younger family members viewed leisure as the means to unwind, reward themselves, and dedicate time to themselves. Rashika, the daughter-in-law of Anasuya, said, “*While doing these activities (leisure)*,* you use the time for yourself. That you do for yourself for which you try to make time…we may say that we do to relax ourselves or to pamper ourselves*.” In contrast, older participants felt that activities benefiting others (family members or social causes) are leisure, as they give them a sense of happiness and satisfaction. Anasuya expressed, “*I get lots of happiness (from social work). I have decided in my heart… till the end… till my body works and moves*,* I will do it*.”

The family members also differed in their perspectives on leisure activities. Older adults described household and productive work as leisure, whereas younger generations considered these activities as work. Siddhesh felt that his grandfather is not able to differentiate between work and leisure, as he has similar sentiments attached to both:

The line becomes very blurred when you love work, right? I cannot say it is leisure because he (Vinayak) loves unwinding down and watching TV…and I do not think I will classify him doing work as a leisurely activity. Therefore, you need work for like engaging you…physical and mentally, so I guess it is more of an engagement of sorts than it is a leisurely activity for him. Therefore, for him, he loves work, but work is still work.

Aditya, son of Pankaj, believed that retirement is all about maintaining a balance between work and leisure and is a time to explore one’s hobbies or long-lost desires to engage in them. He felt that there should be a change in his father’s life after retirement and that he should withdraw from the tedious lifestyle of creating more work for himself. In contrast, his father Pankaj liked engaging in work and thought of it as leisure in his life. He considered engagement in some activities fruitful and pleasurable. Pankaj, who had worked in the corporate sector, continued to occupy his time by supervising his family property and agricultural farms in his native place and said, “*I need to do something… it is not like I come and there is nothing to do. My happiness is continuously being busy*. *This is my relaxation*.”

The differing leisure perspectives among family members influence the support and participation of family members in older adults’ leisure interests. In joint families, family members share the same space and equipment for leisure, and the leisure interests of older adults often conflict with those of other family members. In such situations, older adults tend to prioritize their family members’ interests and wishes over their own and may lose autonomy in their leisure participation.

I mean, for me, it is very annoying. The noise, the content that they (grandparents) watch on TV (television), is bland or nonsensical. Therefore, it truly annoys me. Therefore, I guess it is me I kind of hinder like… I come in between their leisurely activities more than I come across as promoting it. Therefore, like I would find excuses to change the channel. Therefore, I would switch to something that suits my taste, and they will not complain because they truly love me. (Siddhesh, grandson of Vinayak)

#### Family Matters

Older participants also expressed that they were occasionally unable to pursue complex leisure interests, such as travelling to distant places, due to the unavailability of their family members. Consider the case of Vinayak, who felt that travelling to different places was a family affair.

They (family members) do not have time. They do not have holidays… because of work, they do not have leaves. I am free now, but they should also be free…and if time is there then only, we can go (travelling).

Family members’ concerns over older adults’ safety also influence how and where leisure activities are undertaken. Family members were mostly protective and tended to restrict themselves. Veer described, “*A few days ago, my son scolded me as there are many accidents. Therefore, I have started going to nearby parks which are a few minutes away.*” Veer had a preferred route for his daily evening walks that required him to go through a busy area. However, his son was concerned about his safety. Although capable of continuing with his usual route, Veer obliged his son’s suggestion.

#### Environmental Constraints

Participants identified limited infrastructure, finances, and inadequate guidance after retirement as constraints to the leisure participation of older adults.

Municipal cops (co-operations) or local authorities should work to make these (places) available where clubs can be there, and they should be free of cost, so financial things should be considered after retirement. There should be places in the vicinity where retired people can get together. Therefore, there is a lack of guidance after retirement for physical and mental health, I think. (Shirish, son of Veer)

First, we do not have footpaths like real footpaths to walk… the roads are not friendly for someone who is walking and are less friendly for someone who is like not fit. Second, I feel that uh… I think there is no guided program to take care of their health as well. Affordability is also a concern, I guess, for retired people… Some retired elders may not opt for clubs or other places where there are a variety of activities for them… as they may have certain fees… which they may not be able to pay. (Aditya, son of Pankaj)

## Discussion

This study aimed to understand the experiences and perspectives of older adults and family members regarding postretirement leisure in Mumbai, an Indian metropolis. The study involved participants from three generations: older adults, their children, and grandchildren.

### Indian Older Adults’ Perspectives Regarding Leisure after Retirement

The findings of the present study suggest that the leisure activities of older participants included activities that gave them satisfaction. In addition to hobbies, these activities included doing things for others, such as volunteer work and household chores for the family. The older adults valued being productive, preferred activities that utilized their work-related expertise to occupy their free time after retirement and described these activities as leisure for the satisfaction experienced. Such activities are described as serious leisure activities (Stebbins, 2007, 2009; Parker, 2000). These activities were not necessarily directed to the self and were inclined to be altruistic.

Furthermore, older adults’ leisure activities appear more obligatory than free and indistinct from work or daily chores. As reported in other Asian cultures (Walker & Wang, 2008), older adults do not seem to perceive freedom as an essential part of their leisure experience. The concept of leisure being free or non-obligatory while widely accepted is not without debate. Stebbins (2000) posits that obligation is an inherent part of leisure that is experienced to varying degrees, depending on the person’s attitudes towards the activity. This obligation is flexible, as it entails relative freedom to honor commitments, unlike work and personal activities, and is agreeable because it accompanies positive attachment to activities, pleasant memories, and expectations. As a result, leisure may not be experienced as an obligation, as the participant desires to do it anyway.

Another salient finding is that older adults preferred participating in leisure interests with their family members over solitary leisure interests or hobbies. Families play a significant role in the choice of leisure as well as in actual leisure participation. This contrasts with Western studies, where older adults participate in leisure activities alone or in the company of others for the sole purpose of making new friends or developing a sense of belonging (Pereira & Stagnitti, 2008; Ball et al., 2007). This could be attributed to India’s collectivistic roots and values. For example, Nagla (2005) described meal consumption in the Indian context as a leisurely collective ritual, especially in villages, where, in addition to eating, people also talk and enjoy each other’s company.

The older adults in the present study were most likely migrants or children of first-generation migrants to Mumbai in the 1970–1980s. However, most of them continue to live in a joint family system in Mumbai. Even those with nuclear living arrangements preferred to live close to their sons because of the prevalent patriarchal culture. As seen in the present study, family members were encouraging and supportive and tried to give time to their older relatives despite their work commitments. These findings are consistent with those of Jothikaran et al., 2023, suggesting that collectivistic values continue to be upheld even with changing living arrangements. Thus, the meanings ascribed to leisure by urban older retirees appear to differ from those ascribed to conventional Western concepts in terms of time and free will (Purrington & Hickerson, 2013) and individualism (Arai & Pedlar, 2003). This finding questions the prevalent use of the Western definition for leisure studies in India (Badami & Yenagi, 2015; Singh & Misra, 2015; Kadarkar et al., 2016) as suggested by research in other non-Western contexts (Iwasaki et al., 2007).

### Leisure Participation of Retired Indian Older Adults

The leisure activities of the older adults in the present study, i.e., walking, yoga, cooking, watching television, religious activities, and reading, are similar to those in other studies in India (Badami & Yenagi, 2015). The study findings suggest that older participants preferred to engage in leisure interests that they were familiar with either from their preretirement work roles or leisure interests. This finding is consistent with studies in other cultures (Minhat et al., 2013; Agahi et al., 2006; Wanka, 2020).

Socially ascribed gender-based roles appeared to influence pre- and postretirement leisure interests, with women being more involved in home-bound caring or nurturing roles, whereas men preferred outdoor, physical, and productive activities, which is consistent with earlier studies in India (Bhangaokar et al., 2021) and other countries (Jaumot-Pascual et al., 2018). Besides social norms, the leisure engagement of Indian older women belonging to middle and upper class has also been attributed to an enhanced sense of self-actualization and self-management (Tripathi & Samanta, 2023). Although subtle, our study also seems to suggest that older women are more explorative (Jaumot-Pascual et al., 2016, 2018; Bhangaokar et al., 2021; Tripathi & Samanta, 2023).

Both older women and men had work-related leisure interests that could be attributed to similarities in their education and employment. The present study suggests that women’s education, employment, and financial independence may mitigate gender-based differences in leisure participation to some extent. The present study validates earlier recommendations for a gender-based perspective in understanding the leisure participation of older adults (Jaumot-Pascual et al., 2016, 2018).

The findings also identified complex and interrelated personal and contextual factors influencing older adults’ leisure participation. As reported in previous studies (Janke et al., 2006), the older adults in the present study reported that, due to health problems, they had to discontinue, modify or adopt new leisure activities. Adapting to or adopting new leisure activities would require older adults to be flexible and open to other leisure options or resources such as technology and social groups. As reported by family members in the present study, the inflexible attitudes of older adults (Jothikaran et al., 2023; Crombie et al., 2004) may limit their leisure participation, as they grow older, which has detrimental consequences for their health, participation and well-being.

As seen in the study, outdoor leisure activities such as evening walks, travelling, going out for movies or vacations were a concern. These activities occur in complex dynamic environments and require greater physical, cognitive and technological abilities. At one end, older adults are experiencing aging and health-related declines in intrinsic capacities for outdoor leisure and may not have the technical skills required for planning, online transactions, etc. At the other end, the environment in a metropolis such as Mumbai, which is experiencing significant urbanization, is becoming increasingly challenging and unsafe with increasing population, traffic and digitalization of public transport (Mumbai Traffic Control Branch & Bloomberg Philanthropies Initiative for Global Road Safety, 2020; Pandya, 2023). As a result, older participants were concerned about their safety, were not confident in their ability to pursue outdoor leisure activities independently, and needed their family members’ assistance (Crombie et al., 2004; Śniadek & Zajadacz, 2010),

Furthermore, owing to their collectivist values, Indian older adults expect their children to look after them (Gupta & Hershey, 2016; Jothikaran et al., 2023; Chadda & Deb, 2013) and may not feel the need to be independent. In addition to safety, older adults value the company of their family members in family leisure activities, and their leisure experiences are intricately linked to their families. Irrespective of living arrangements, older adults’ leisure experience and participation depend on the availability of their family members. However, as seen in the present study, family members were not always available due to work demands that limited older adults’ leisure participation and satisfaction derived from leisure.

Additionally, older participants’ perspectives regarding leisure appeared to differ from those of their younger family members, who appeared to embrace more Western leisure concepts. This finding is in line with Twenge et al. (2010), who reported that leisure values increased steadily over generations, as the younger generation values work‒life balance more than did the older generations. Intergenerational differences may influence family members’ support for and participation in the leisure interests of older adults and the utilization of shared resources for leisure. In modest-sized apartments, family members need to share the same space and equipment, such as television. Older adults may not have personal space and privacy for leisure. In such situations, older adults may not assert themselves or may prioritize their family members’ needs, as seen in the present study.

Another factor could be the changing authority structure in Indian families. In the traditional patriarchal authority structure, the older person is considered the head of the family and makes decisions (Singh, 2009; Chakravorty et al., 2021). The present study suggests that this structure is changing. While some families were collaborative, in others, children appeared to have more authority. The older participant *scolded* by his son felt obliged to listen to his son and did not assert his ability to walk safely in the community. This shift in the balance of power between older parents and adult children may occur despite the former’s financial independence (Chakravorty et al., 2021). Altered family dynamics following retirement owing to changes in attitudes of the retiree and family members. (Raju, 2011) may further impact older adults’ autonomy in leisure participation. However, recent studies have reported changing trends in older Indians’ leisure engagement. With increased financial independence and consumerism, older especially from upper middle class are demonstrating increased autonomy, self-reliance and resourcefulness in their leisure choices and engagement (Samanta, 2018; Tripathi & Samanta, 2023).

The present study identified environmental challenges such as limited access to and affordability of community resources, especially for outdoor physical leisure activities, which is consistent with earlier studies (DiPietro, 2001; Badami & Yenagi, 2015; Crombie et al., 2004; Paillard-Borg et al., 2009). In Indian cities, poor roads, inadequate transport, and social and attitudinal barriers affecting community mobility (Ramachandran & Dsouza, 2016, 2018) continue to be prevalent problems that prevent older adults from walking, which is a common and feasible leisure activity. The study also suggests the limited availability of retirement transition programs for older adults.

### Implications

The present study is a preliminary study providing insights into the personal and contextual factors influencing the leisure experiences of older retirees in an Indian metropolis. Considering the importance of leisure to support health and well-being following retirement, the study findings may support the provision of contextually relevant leisure interventions for Indian older adults. Professionals providing leisure interventions need to be mindful of different perspectives about leisure within and between cultures and generations and should be wary of generic lists of leisure activities developed from Western literature (Purrington & Hickerson, 2013). Personal and family influences may be more accessible and amenable to intervention and should be prioritized. Therefore, understanding and addressing the unique personal factors influencing leisure participation, especially older adults’ leisure, personality traits, attitudes towards leisure, and technological readiness, is important. Leisure interventions may need to consider living arrangements, familial influences and the involvement of family members. Intergenerational differences and family dynamics may need to be negotiated if they pose barriers. Older adults need to be made aware of the changing times and the importance of increasing their self-reliance for leisure and other daily living activities. While such sensitization could be performed through informal sources such as family and media, formal sources such as retirement transition programs are probably more feasible and acceptable for retiring older adults. Leisure education should be incorporated into retirement preparation programs. (Kleiber & Linde, 2014). The study findings support ongoing advocacy for senior-friendly, accessible and safe transport facilities, outdoor environments and community resources such as public parks (Levinger et al., 2018; Ramachandran & Dsouza, 2016, 2018) in urban planning, especially in developing Tier-II cities identified for the smart city missions of the Indian government (Ministry of Housing and Urban Affairs, 2019).

### Strengths and Limitations

The participants were purposively selected, and the study had a homogenous sample. The findings are transferable to older adults with similar characteristics, i.e., urban dwelling educated older retirees of middle and upper SES. The authors’ familiarity with the participants’ linguistic and cultural context and phenomenon supported a better understanding and interpretation of their narratives. The study also has limitations. Owing to resource constraints, it was difficult to perform follow-up interviews and back-translation for all the interviews conducted in Indian languages that may have impacted the findings. The authors also appreciate that their conceptions and beliefs may not permit complete access and understanding of the participants’ subjective experiences.

## Conclusion

This study provides insights into older retirees’ experiences and perspectives of leisure in an Indian metropolis. The findings suggest that the meanings ascribed to leisure by Indian older adults are different from the conventional Western concept of leisure. The study elucidates personal and contextual factors influencing older retirees’ leisure choices and participation, especially familial influences and environmental constraints. The study findings would inform further research on leisure in the Indian context and facilitate the provision of contextually relevant interventions to support meaningful leisure participation in older adults and healthy aging.

## Data Availability

Data will be made available on request.
